# Menthol cigarette alternative products: Awareness, use, and substitution intentions among adults who smoke menthol cigarettes

**DOI:** 10.1016/j.addbeh.2025.108470

**Published:** 2025-08-27

**Authors:** Christina N. Kyriakos, Grace Kong, Akshika Sharma, Meghan E. Morean, Sakinah C. Suttiratana, Krysten W. Bold

**Affiliations:** a Department of Psychiatry, Yale School of Medicine, Yale University, New Haven, CT, USA; b Department of General Internal Medicine, Yale School of Medicine, Yale University, New Haven, CT, USA

**Keywords:** Flavor accessories, Menthol cigarette alternatives, Menthol ban, Menthol cigarettes

## Abstract

**Background:**

Menthol cigarette alternative products (e.g., menthol accessories, non-menthol cooling cigarettes) have entered the market and may bypass menthol cigarette restrictions. Understanding awareness, use, and substitution intentions if menthol cigarette sales are prohibited is important for assessing their potential to undermine the public health benefit of such policies.

**Methods:**

We conducted a cross-sectional survey in 2023 of N = 116 Connecticut adults who smoked menthol cigarettes (mean age = 33.4, 50.9 % female, 50.9 % Black, 13.8 % Hispanic). We assessed awareness and ever use of menthol cigarette alternative products including menthol accessories (i.e., menthol filter capsules, sprays, cards, filter tips) and non-menthol cooling cigarettes with synthetic coolants. We measured substitution intentions to use each product if menthol cigarettes were restricted. Logistic regression models examined associations between sociodemographic and smoking characteristics and outcomes of awareness, ever use, and substitution intentions.

**Results:**

Overall, 66.4% were aware of at least one type of menthol accessory, 41.4% had used one, and 80.2% reported substitution intentions if menthol cigarettes were restricted. Moreover, 39.6% were aware of non-menthol cooling cigarettes, 25.9% had used them, and 56.0% reported substitution intentions. Younger age was associated with awareness of menthol accessories and non-menthol cooling cigarettes. Higher nicotine dependence was associated with awareness, ever use, and substitution intentions across all products.

**Conclusions:**

We found considerable awareness, ever use, and substitution intentions of menthol cigarette alternative products among adults who smoke menthol cigarettes. Findings underscore the importance of comprehensive menthol cigarette restrictions that cover accessories and non-menthol cooling cigarettes to maximize public health.

## 1. Introduction

Menthol cigarettes remain a large public health problem due to menthol’s role in increasing cigarette appeal and facilitating initiation and progression to regular use. (Villanti et al., 2019; World Health Organization, 2016) A number of countries and jurisdictions have enacted policies prohibiting the sale of menthol and other flavored cigarettes. (Erinoso et al., 2021) Menthol cigarette restrictions have been shown to reduce menthol cigarette use and increase quitting among those who previously smoked menthol (vs. non-menthol). (East et al., 2022; Fong et al., 2022; Chung-Hall et al., 2021; Chaiton et al., 2021; Kyriakos et al., 2022) A systematic review found that approximately one-quarter of people who smoke menthol cigarettes quit after a menthol cigarette restriction. (Mills et al., 2024) However, one-quarter report continuing to smoke menthol cigarettes post-restriction. (Mills et al., 2024; Stoklosa, 2019) Illicit purchasing has not increased in places that have enacted menthol cigarette restrictions, such as Canada, the European Union (EU), or the United Kingdom (UK). (Stoklosa, 2019; Chung-Hall et al., 2023; Kyriakos et al., 2023) Rather, continued menthol cigarette use post-restriction may be driven by use of products that circumvent the restriction (i.e., flavor accessories that add flavor to cigarettes, as well as cigarettes that have been re-formulated with other chemicals that still have a cooling effect). (Hiscock et al., 2020; Brink et al., 2022; Jabba et al., 2023) We hereby refer to these as “menthol cigarette alternative products”.

Flavor accessories, which are distinctly separate components that can be used to add flavor to cigarettes, come in a variety of forms (e.g., cards, capsules, filters, drops), and are available in many countries. (Havermans et al., 2022) While the market introduction and advertising of these products have been primarily observed alongside menthol restrictions, flavor accessories have also entered the market in jurisdictions where a policy is not in place. (Havermans, et al., 2024) This includes the United States (US), where no federal policy exists although restrictions on the sale of menthol cigarettes have been enacted in some states (i.e., Massachusetts and California) and municipalities.

Another menthol cigarette alternative product is cigarettes that are marketed as “non-menthol,” but mimic characteristics of menthol cigarettes. (Meza et al., 2025) For example, in response to the restriction on menthol cigarettes in California and Massachusetts, “non-menthol” cooling cigarettes that contain synthetic coolants and other flavor chemicals emerged in the market (e.g., Camel Crisp Non-Menthol, Newport Non-Menthol). (Jabba et al., 2023; Page et al., 2023) Similar products have also been observed after menthol restrictions in the EU and UK. (Hiscock et al., 2020) Despite the availability of flavor accessories and non-menthol cooling cigarettes in many places, only a few studies have examined awareness and use of menthol cigarette alternative products. (Kyriakos et al., 2023; Havermans et al., 2022; Chaiton et al., 2021; Kyriakos et al., 2021; Choi et al., 2025) Only one study, which examined the epidemiology of non-menthol cooling cigarettes, was conducted in the US, (Choi et al., 2025) with no known US population-based studies on flavor accessories.

Understanding the landscape of menthol cigarette alternative products, including both non-menthol cooling cigarettes and menthol accessories, is important to inform future regulations. (US Food and Drug Administration, 2022) The goal of a menthol cigarette ban is to reduce smoking and its associated harms. However, simply banning menthol cigarettes without addressing separate accessories that can add menthol flavor to unflavored cigarettes or cigarettes designed to mimic menthol’s sensory effects, risks undermining this public health objective. Availability and use of menthol cigarette alternative products may reduce the effectiveness of a menthol restriction if adults who smoke menthol cigarettes switch to using these products to continue smoking. Therefore, it is also critical to understand the extent to which these products may potentially be used as substitutes in the context of a menthol cigarette restriction. Menthol cigarette alternative products can also enhance the appeal of tobacco and elicit sensory effects comparable to menthol cigarettes, (Jabba et al., 2023; Page et al., 2023) highlighting the need for a greater understanding of their awareness and use.

This study therefore aimed to examine awareness and ever use of menthol cigarette alternative products, as well as substitution intentions if there was a restriction on the sale of menthol cigarettes among adults who smoke menthol cigarettes. Additionally, this study aimed to assess how these outcomes vary across key sociodemographic and smoking behaviors, such as age, race, sex, and nicotine dependence, given their established association with menthol cigarette and other tobacco product use. (Goodwin et al., 2023) We focused on adults who smoke menthol cigarettes and reside in Connecticut. Connecticut offers a unique opportunity to study product awareness and availability among people who smoke since menthol cigarettes are sold legally despite recent state and local policy discussions, in contrast to neighboring state Massachusetts, where the sales of menthol and flavored tobacco products are prohibited. This circumstance may offer insights into spillover knowledge and dissemination of menthol cigarette alternative products.

## 2. Methods

### Study design.

A cross-sectional online survey was conducted among 149 Connecticut adults who smoked cigarettes in the past 30 days. Study eligibility included providing online consent, being at least 21 years old, residing in Connecticut (verified by address and zip code, latitude, longitude), reporting smoking cigarettes in the past 30 days, and passing attention checks. Screening quotas were set to recruit at least 40 % who identified as Black or Hispanic/Latinx to better reflect the population of adults who smoke and are most vulnerable to tobacco-related health disparities. Participants were recruited through online social media postings, flyers, community listservs, and direct email invitations to lists of study participants who agreed to be contacted about ongoing studies. For this study, the analytic sample was restricted to those who reported usually smoking menthol cigarettes or smoking both menthol and non-menthol cigarettes (N = 116).

### Measures.

#### Awareness and ever use of menthol cigarette alternative products and substitution intentions to use these products if menthol cigarettes were restricted.

The survey described several menthol cigarette alternative products, including menthol accessories and non-menthol cooling cigarettes, using brief descriptions and pictures (Supplementary Material). The following menthol accessories were described— 1) menthol filter capsules (e.g., crush balls, flavor popping beads), 2) menthol spray for cigarettes, 3) menthol flavor cards for cigarettes, and 4) menthol filter tips for roll your own cigarettes. The survey also described non-menthol cooling cigarettes, with images depicting Newport Non-Menthol and Camel Crisp Non-Menthol brands as examples.

The survey assessed awareness of each product as ever having heard of the product (yes, no). Among those who reported awareness, ever use of the product was assessed (yes, no). Those who had never heard of the product were categorized as having never used. Substitution intentions if menthol cigarettes were restricted was examined with the question, “*If menthol cigarettes were no longer available, would you use [product] as a replacement?”* with response options of definitely not, probably not, probably yes, definitely yes. We operationalized “probably yes” or “definitely yes” as likely to use as a substitute and “probably no” or “definitely no” as unlikely to use as a substitute.

A composite variable, ‘at least one type of menthol accessory’, was derived from reporting “yes” to any of the following four products: menthol filter capsules, menthol spray, menthol flavor cards, or menthol filter tips, for the respective outcomes related to awareness, ever use, and substitution intentions.

#### Sociodemographics and smoking behaviors.

Participants reported on age (in years), biological sex (female; male), annual household income (operationalized as ≤$50,000 and >$50,000), (Lee et al., 2024) Hispanic ethnicity (yes; no), and race (select all that apply: American Indian or Alaska Native; Asian; Black or African American; Native Hawaiian; Pacific Islander; White; Other). Respondents were categorized as ‘Black’ if they selected “Black or African American” regardless of any other race categories selected and ‘Non-Black’ if they did not select “Black or African American”. These categories were selected due to the small sample of respondents that selected a race other than White or Black/African American (n = 5) and due to Black identity being a variable of interest in the context of a potential restriction on menthol cigarettes due to the disproportionately higher use of menthol cigarettes among those identifying as Black in the US. (Cheng et al., 2024) Nicotine dependence was assessed using the 4-item Patient-Reported Outcomes Measurement Information System (PROMIS) Smoking Nicotine Dependence for All Smokers-Short Form 4a Test for Nicotine Dependence (5-point Likert scale; total possible raw score range: 4–20). (Shadel et al., 2014) Use of another tobacco product in the past 30 days was a covariate that was assessed based on reported use of at least one of the following: e-cigarette; cigar, cigarillo, or little cigar; hookah, smokeless tobacco (chew, snuff or dip); nicotine pouches (all assessed as yes, no).

#### Analysis.

All analyses were conducted in Stata/MP 18.0. Descriptive statistics were run to examine rates of awareness, ever use, and substitution intentions for (1) the composite variable of ever using at least one type of menthol accessory, (2) each individual menthol accessory (capsules, filter tips, card, spray), and (3) non-menthol cooling cigarettes.

We then ran separate logistic regression models for awareness, ever use, and substitution intentions each for: (1) at least one type of menthol accessory and (2) non-menthol cooling cigarettes. To examine effects by sociodemographic characteristics and smoking behaviors, the following variables were included in all models as independent variables: sex (female vs. male), Hispanic ethnicity (yes vs. no), race (Black vs. Non-Black), age, household income (≤$50,000 vs. >$50,000), (Lee et al., 2024) nicotine dependence, and use of another tobacco product in the past 30 days (yes vs. no). The alpha level for statistical significance was set at 0.05. (Doi et al., 2022).

## 3. Results

### Sample characteristics.

Among adults who smoked menthol cigarettes (n = 116), 50.9 % were male, 13.8 % identified as Hispanic, 50.9 % identified as Black, the mean age was 33.4 years (SD = 10.07), 72.4 % reported a household income of ≤$50,000, the mean raw total PROMIS score for nicotine dependence was 11.3 (SD = 4.67), and 73.3 % reported using another tobacco product in the past 30 days (Table 1).

### Awareness of menthol cigarette alternative products.

Overall, 66.4 % (95 % CI: 52.7–74.4 %) had heard of at least one type of menthol accessory (Table 2). Awareness was highest for menthol filter tips (44.8 %, 35.9–54.0 %) and menthol filter capsules (43.1 %, 34.3–52.3 %), followed by menthol cards (28.4 %, 20.8–37.4 %) and menthol sprays (25.0 %, 17.9–33.8 %). Those who reported greater nicotine dependence (aOR = 1.18, 1.04–1.34, *p* = 0.011) were more likely to be aware of at least one type of menthol accessory, while older individuals were less likely to be aware of at least one type of menthol accessory (aOR = 0.95, 95 % CI: 0.90–1.00) (Table 3).

In total, 39.6 % (31.1–48.9 %) were aware of non-menthol cooling cigarettes (Table 2). Similar findings were observed as with menthol accessories where those with greater nicotine dependence (aOR = 1.19, 1.06–1.34, *p* = 0.003) were also more likely to be aware of non-menthol cooling cigarettes and older individuals less likely to be aware of non-menthol cooling cigarettes (aOR = 0.94, 0.89–0.99, *p* = 0.032) (Table 4).

### Ever use of menthol cigarette alternative products.

Among all participants, 41.4 % (32.7–50.6 %) reported ever trying a menthol accessory, with rates of trying highest for menthol filter capsules (29.3 %, 21.7–38.3 %) and menthol filter tips (24.1 %, 17.1–32.8 %) and lower for menthol cards (17.2 %, 11.3, 25.3 %) and menthol spray (16.4 %, 10.6–24.3 %) (Table 2). Those identifying as Black compared to Non-Black (aOR = 0.36, 0.14–0.97, *p* = 0.042) and those with a household income ≤$50,000 compared to those with income of >$50,000 (aOR = 0.28, 0.08, 0.92, p = 0.036) had lower odds of ever using at least one type of menthol accessory (Table 3). Those with greater nicotine dependence were more likely to have ever used at least one type of menthol accessory (aOR = 1.22, 1.08–1.39, *p* = 0.002).

Moreover, 25.9 % (18.6–34.7 %) had ever used non-menthol cooling cigarettes (Table 2). Females were less likely to have ever used non-menthol cooling cigarettes compared to males (aOR = 0.21, 0.06–0.79, *p* = 0.021), while those reporting greater nicotine dependence were more likely to have ever tried non-menthol cooling cigarettes (aOR = 1.24, 1.08–1.43, *p* = 0.002) (Table 4).

### Substitution intentions with menthol cigarette alternative products if menthol cigarettes were restricted.

When considering a hypothetical ban on menthol cigarettes, 80.2 % (71.8–86.5 %) reported intentions to use at least one type of menthol accessory as a substitute for menthol cigarettes (Table 2). Menthol filter capsules were the most-endorsed substitute (63.8 %, 54.6–72.1 %), followed by menthol filter tips (52.6 %, 43.4–61.6 %), menthol cards (51.7 %, 42.6–60.7 %), and menthol spray (41.4 %, 32.7–50.6 %). Greater nicotine dependence was associated with intentions to use at least one type of menthol accessory as a substitute for menthol cigarettes (aOR = 1.15, 1.01–1.34, *p* = 0.039 (Table 3).

In addition, 56.0 % (46.8–64.9 %) reported intentions to use non-menthol cooling cigarettes as a substitute if menthol cigarettes were no longer sold (Table 2). Greater nicotine dependence was also associated with intentions to use non-menthol cooling cigarettes as a substitute (aOR = 1.23, 1.09–1.38, *p* = 0.001) (Table 3).

## 4. Discussion

This study is one of the first to examine use of menthol cigarette alternative products in a US sample. We found that moderate proportions of adults who smoke menthol cigarettes were aware of and had ever used at least one type of menthol accessory and non-menthol cooling cigarettes. The majority reported that they would likely use at least one type of menthol accessory and half would use non-menthol cooling cigarettes as a substitute if menthol cigarettes were restricted. Menthol filter capsules and menthol filter tips were the accessory types that were most common and likely to be used as substitutes for menthol cigarettes.

Our findings that menthol cigarette alternative products are familiar and used are concerning given that the products are relatively new. These products have features known to increase the appeal of tobacco, including enhanced flavoring, cooling sensory effects, and novelty. (O’Connor, 2023) Moreover, since behavioral intentions can increase the likelihood of actual behavior, the observed high endorsement of using menthol cigarette alternative products as a substitute suggests that those who currently smoke menthol cigarettes may switch to these products rather than quit smoking if the sale of menthol cigarettes is restricted. (Glanz et al., 2015) Use of these products prior to a restriction may further facilitate their use as substitutes and decrease the public health benefit of a menthol cigarette policy if these products are not regulated.

The observation that menthol capsules and filter tips were the most commonly known and used menthol accessory types and the most likely substitute for menthol cigarettes aligns with previous research. In a study examining online retail availability of flavor accessories across eight countries, including the US, flavor capsules were the most commonly available product and had the largest number of unique brands and manufacturers. (Havermans, et al., 2024) The proliferation of videos promoting flavor capsule accessories has also been observed on social media (TikTok). (Dobbs et al., 2024) The popularity of menthol capsule accessories may further be attributed to their ability to replicate flavor capsule cigarettes, which contain pre-installed capsule(s) in the filter rather than requiring the consumer to insert them. Flavor capsule cigarettes are appealing, particularly among young people. (Kyriakos et al., 2021) In line with our study, menthol filter accessories were also the most commonly reported accessories used after a menthol cigarette restriction in Canada (Kyriakos et al., 2021), England (Kyriakos et al., 2021), and the Netherlands. (Kyriakos et al., 2023) Further, in England, an observed increase in the predominant use of roll-your-own tobacco among those smoking menthol cigarettes from before to after the menthol restriction has been attributed to the availability of menthol filter accessories. (Buss et al., 2024).

Our finding that 80 % reported intentions to use any type of menthol accessory if menthol cigarettes were restricted further highlights the potential for these products to undermine a menthol restriction if these products are not regulated. However, our figure is much higher than what was observed in a study conducted in Ontario, Canada, in which Chaiton et al. (2018) found that prior to the menthol ban, only 1 % of individuals who smoked menthol cigarettes expected to use menthol accessories after the ban. Shortly after the ban 14 % reported actually doing this and none reported long-term plans to use menthol accessories. (Chaiton et al., 2018) These differences may be reflective of changes in the flavor accessories market since the time of that study or contextual factors, such as population and country differences.

Unlike menthol accessories which can be purchased online in Connecticut, non-menthol cooling cigarettes, to our knowledge, were only marketed and sold in the US in Massachusetts and California at the time of this study. Nevertheless, our finding that 40 % of adults who smoked menthol cigarettes were aware of non-menthol cooling cigarettes is comparable to a national study that examined this (37 %), which included both states with and without a menthol ban. (Choi et al., 2025) We also found that a quarter of adults who smoked menthol cigarettes had ever used non-menthol cooling cigarettes. The relatively high awareness and ever use of these products may be due to Connecticut’s close proximity to Massachusetts, allowing consumers to purchase them during transit. (Jabba et al., 2023) It is further plausible that awareness of these products has been influenced by possible increased media coverage and social media discussions surrounding the menthol restrictions in Massachusetts and California, as well as the proposed US federal restriction (Feng et al., 2024). This could further explain why awareness was higher among younger adults, who tend to use social media more frequently. (Hruska and Maresova, 2020) It is also possible that participants misreported having tried non-menthol cooling cigarettes, given that the packaging and messaging are nearly identical to regular menthol cigarettes. (Meza et al., 2025).

Our finding that non-menthol cooling cigarettes are a likely substitute for menthol cigarettes in the context of a menthol ban is consistent with a previous study using a virtual store experiment. (Guillory et al., 2020) Guillory et al. (2020) found that those who smoked menthol cigarettes made fewer cigarette purchases when menthol cigarettes were restricted; however this reduction was not observed when ‘green replacement’ cigarettes were available, as they were more likely to switch to a different brand. (Guillory et al., 2020) Findings suggest that these products may undermine a menthol cigarette restriction by encouraging continued cigarette use.

Certain populations, such as Black individuals, younger people, as well as those with high nicotine dependence may benefit most from a menthol restriction in the US. However, these groups are also especially vulnerable to continued cigarette smoking after such a policy is implemented. (Cheng et al., 2024) As such, our study examined how outcomes on menthol cigarette alternative products varied across these groups. We found that higher nicotine dependence was consistently associated with awareness, ever use, and substitution intentions of menthol accessories and non-menthol cooling cigarettes, highlighting how menthol cigarette alternative products may especially encourage continued use rather than quitting in this group. Our finding that younger age was associated with greater awareness of menthol accessories and non-menthol cooling cigarettes may be due to greater exposure of these products through possible marketing channels, such as social media, as previously described. While our study did not find that younger age was associated with ever use of menthol cigarette alternative products, the potential for young people to use these products is high. Past studies have reported youth and young adults to be early adopters of various tobacco products, with flavors playing a significant role in their initiation and use. (Villanti et al., 2019; Patel et al., 2016; Kasza et al., 2020) Further, in countries with a restriction on menthol cigarettes, use of menthol accessories is consistently associated with younger age. (Kyriakos et al., 2023; Chaiton et al., 2021; Kyriakos et al., 2021; Pauwels et al., 2022).

While it is promising that we did not observe disproportionately higher awareness, ever use, and substitution intentions across menthol cigarette alternative products among those identifying as Black compared to non-Black, the high use of menthol cigarettes in this population (Goodwin et al., 2023) warrants continued monitoring. In England and Canada, where disparities in menthol cigarette use by race/ethnicity were not observed prior to the menthol restriction, youth identifying as Black and other race/ethnicities had significantly greater odds of using menthol accessories compared to their White peers after the restriction. (Kyriakos et al., 2021) It is possible that disparities in use of menthol cigarette alternative products may emerge if more concentrated marketing efforts take effect. The tobacco industry may use the same marketing tactics historically used for menthol cigarettes to target Black and younger populations in the US with new menthol cigarette alternative products. (Anderson, 2011) Understanding the current and continued marketing landscape of these products is needed.

This study has some limitations. First, participants were recruited using convenience sampling, which may introduce selection bias and limit the generalizability of findings. However, our sample characteristics are generally comparable to nationally representative data from the International Tobacco Control (ITC) project among adults who smoke menthol cigarettes in the US, except that in our study we oversampled Black adults and we had a higher percentage of Hispanic participants (our study vs ITC study: female: 49.1 % vs 50.8 %, Hispanic: 13.8 % vs 8.4 %, Black: 50.9 % vs 26.3 %, Income: 72.4 % ≤$50,000 vs 64.0 % <$45,000). (Driezen et al., 2024) Nevertheless, future studies should examine menthol cigarette alternative products in a larger and nationally representative sample. Second, the limited sample size reduces statistical power, potentially limiting the ability to detect certain associations. Additionally, the study may not capture the full range of menthol cigarette alternative products available, nor does it account for the use of other types of flavored accessories beyond menthol. Our study also does not provide insight on where participants learned about and accessed menthol cigarette alternative products, an area that needs to be further explored. As mentioned, participants could have misreported use of non-menthol cooling cigarettes given their similarities in packaging with menthol cigarettes, as well as misreported use of menthol accessories. However, we provided descriptions and images of the specific products to limit misreporting (Supplementary Material). It is also possible that participants were inaccurate in estimating their future substitution behavioral intentions in response to a menthol ban, as has been observed in other studies. (Chaiton et al., 2018) Despite these limitations, this is one of the first studies to examine the use of menthol cigarette alternative products in the US, providing valuable insights into current use trends and the potential for these products to be used as a substitute in a future restriction on the sale of menthol cigarettes.

Our study has important regulatory implications and identifies key research gaps. The strong potential for menthol cigarette alternative products to serve as substitutes if menthol cigarettes are restricted, as demonstrated in our study, underscores how these products could weaken the effectiveness of menthol cigarette restrictions if policies fail to address them. In May 2022, the US Food and Drug Administration (FDA) proposed a product standard to prohibit menthol as a characterizing flavor in cigarettes nationwide. The proposed standard covered flavor accessories by specifying that the rule would “prohibit the use of menthol as a characterizing flavor in cigarettes and cigarette components and parts, including those that are sold separately to consumers”. (US Food and Drug Administration, 2022) Further, non-menthol cooling cigarettes would also be restricted if they were determined to have a characterizing flavor based on the multisensory experience (i.e., cooling sensations). (US Food and Drug Administration, 2022) However, this policy was withdrawn in January 2025 (although a related lawsuit remains pending in federal court) (Public Health Law Center, 2024) and future menthol policies may benefit from applying lessons learned from other countries and some US jurisdictions. A comprehensive menthol restriction may be most effective at reducing smoking if it covers flavor accessories, as well as additives with flavor and/or sensory properties (i. e., cooling) at any level, thereby covering non-menthol cooling cigarettes. (Chung-Hall et al., 2023; Kyriakos et al., 2023) In addition, explicitly prohibiting marketing and labelling of products as having menthol-like qualities may further support regulatory efforts. For example, some non-menthol cooling cigarette products were determined to violate the California flavor ban law on the premise that the packaging and promotional material still communicate that the product has a characterizing flavor. (Public Health Law Center, 2023) In response to these enforcement challenges, the legislation was strengthened to expand the definition of characterizing flavor to include “cooling sensation”. (No, 2024) The law was also amended to create a list of all tobacco products that are unflavored and legal to sell in California. (No, 2024) Potential future policies may be most impactful if they apply a similarly comprehensive approach to minimize regulatory loopholes of menthol cigarette alternative products. Moreover, a nationwide ban may be more effective than state-level bans by providing uniform coverage and reducing opportunities for circumvention through cross-border purchasing. Lastly, non-menthol cooling cigarette brands may be reviewed by the FDA as Substantially Equivalent (SE) to menthol cigarettes or considered exempt from SE, allowing them to enter or remain on the market without undergoing full premarket review. A future federal policy may benefit from explicitly stating that a menthol ban applies to these products and from applying stricter premarket review requirements.

Our finding that menthol cigarette alternative products are being used even in a state without a menthol restriction highlights the need for monitoring their use over time and across diverse populations and policy environments. Understanding the use and addiction potential of menthol cigarette alternatives, as well as their influence on smoking behaviors, appeal, and user motivations, is critical to assessing their public health impact, particularly among priority populations. Additional research is needed to evaluate whether these products are acceptable substitutes for menthol cigarettes and how they compare to switching to other products (e.g., menthol e-cigarettes). Monitoring marketing tactics is also important for identifying the channels and strategies used to promote menthol cigarette alternative products.

## 5. Conclusions

Among a sample of adults who smoke menthol cigarettes, awareness and ever use of menthol cigarette alternative products, including menthol accessories and non-menthol cooling cigarettes, was substantial. The majority of participants reported that they would likely use these products as a substitute if menthol cigarettes were restricted. Findings suggest that non-menthol cooling cigarettes and menthol accessories have the potential to undermine menthol restrictions that do not comprehensively cover these alternative products.

## Supplementary Material

MMC1

## Figures and Tables

**Table 1:** Sample characteristics, among adults in Connecticut who smoke menthol cigarettes (N = 116).

Characteristics	n	% or Mean	95 %CI or SD
**Sex**			
Male	59	50.9 %	(41.7, 59.9)
Female	57	49.1 %	(40.1, 58.3)
**Hispanic**			
Yes	16	13.8 %	(78.6, 91.4)
No	100	86.25	(78.6, 91.4)
**Race**			
Black	59	50.9 %	(41.7, 59.9)
Non-Black	57	49.1 %	(40.1, 58.3)
**Age (years)**	116	33.4	10.07
**Household income**			
≤$50,000	84	72.4 %	(63.5, 79.8)
>$50,000	32	27.6 %	(20.1, 36.5)
**Nicotine dependence (PROMIS total score)**	116	11.3	4.67
Used another tobacco product* **in past 30 days**			
Yes	85	73.3 %	(64.4, 80.6)
No	31	26.7 %	(19.4, 35.6)

* E-cigarette, cigar, hookah, smokeless tobacco, nicotine pouch.

CI= Confidence Interval; SD= Standard Deviation

**Table 2:** Awareness, ever use, and substitution intentions of menthol cigarette alternative products, among adults from Connecticut who use menthol cigarettes (N = 116).

	Awareness			Ever use			Likely to use as a substitute if menthol cigarettes were restricted		
Type of menthol cigarette alternative product	n	%	95 %CI	n	%	95 %CI	n	%	95 %CI
At least one type of menthol accessory	77	66.4	(57.2, 74.4)	48	41.4	(32.7, 50.6)	93	80.2	(71.8, 86.5)
Menthol filter capsules	50	43.1	(34.3, 52.3)	34	29.3	(21.7, 38.3)	74	63.8	(54.6, 72.1)
Menthol filter tips	52	44.8	(35.9, 54.0)	28	24.1	(17.1, 32.8)	61	52.6	(43.4, 61.6)
Menthol cards	33	28.4	(20.8, 37.4)	20	17.2	(11.3, 25.3)	60	51.7	(42.6, 60.7)
Menthol spray	29	25.0	(17.9, 33.8)	19	16.4	(10.6, 24.3)	48	41.4	(32.7, 50.6)
Non-menthol cooling cigarettes	46	39.6	(31.1, 48.9)	30	25.9	(18.6, 34.7)	65	56.0	(46.8, 64.9)

CI= Confidence Interval.

**Table 3:** Association of characteristics with awareness, ever use, and substitution intentions of menthol flavor accessories (flavor capsules, spray, cards, or filter tips), among adults from Connecticut who use menthol cigarettes (N = 116).

	Aware of at least one type of menthol accessory					Ever used at least one type of menthol accessory					Likely to use at least one type of menthol accessory as a substitute if menthol cigarettes were restricted				
	n	%	aOR	95 %CI	p-value	n	%	aOR	95 %CI	p-value	n	%	aOR	95 %CI	p-value
**Sex**															
Female	32	56.1	1.09	(0.42, 2.87)	0.853	12	21.0	0.42	(0.16, 1.11)	0.080	44	77.2	1.66	(0.53, 5.21)	0.388
Male	45	76.3	1.00			36	61.0	1.00			49	83.0	1.00		
**Hispanic**															
Yes	13	81.2	2.31	(0.54, 9.91)	0.259	8	50.0	1.34	(0.39, 4.62)	0.645	12	75.0	0.47	(0.11, 1.92)	0.291
No	64	64.0	1.00			40	40.0	1.00			81	81.0	1.00		
**Race**															
Black	33	55.9	0.48	(0.19, 1.19)	0.112	17	28.8	0.36	(0.14, 0.97)	**0.042**	44	74.6	0.47	(0.16, 1.40)	0.178
Non-Black	44	77.2	1.00			31	54.4	1.00			49	86.0	1.00		
**Age**															
Mean (SD)	77	32.6 (8.77)	0.95	(0.90, 1.00)	**0.038**	48	32.5 (7.36)	0.96	(0.91, 1.02)	0.181	93	33.9 (9.50)	1.00	(0.95, 1.05)	0.966
**Household income**															
≤$50,000	49	58.3	0.30	(0.08, 1.10)	0.069	25	29.8	0.28	(0.08, 0.92)	**0.036**	65	77.4	0.63	(0.17, 2.37)	0.492
>$50,000	28	87.5	1.00			23	71.9	1.00			28	87.5	1.00		
**Nicotine dependence (PROMIS total score)**															
Mean (SD)	77	12.3 (4.47)	1.18	(1.04, 1.34)	**0.011**	48	13.7 (4.04)	1.22	(1.08, 1.39)	**0.002**	93	12.0 (4.67)	1.16	(1.01, 1.34)	**0.039**
Used another tobacco product* **in past 30 days**															
Yes	61	71.8	2.41	(0.85, 6.83)	0.097	41	48.2	2.90	(0.84, 10.04)	0.092	73	85.9	2.79	(0.91, 8.53)	0.072
No	24	51.6	1.00			7	22.6	1.00			20	64.5	1.00		

* E-cigarette, cigar, hookah, smokeless tobacco, nicotine pouch.

aOR = Adjusted Odd’s Ratio; CI= Confidence Interval, SD= Standard Deviation **Bolded** = p-value < 0.05.

**Table 4:** Association of characteristics with awareness, ever use, and substitution intentions of non-menthol cooling cigarettes, among adults from Connecticut who use menthol cigarettes (N = 116).

	Aware of non-menthol cooling cigarettes (N = 46)					Ever used non-menthol cooling cigarettes (N = 30)					Likely to use non-menthol cooling cigarettes as a substitute if menthol cigarettes were restricted (N = 65)				
	n	%	aOR	95 %CI	p-value	n	%	aOR	95 %CI	p-value	n	%	aOR	95 %CI	p-value
**Sex**															
Female	13	22.8	0.58	(0.22, 1.51)	0.264	4	7.1	0.21	(0.06, 0.79)	**0.021**	26	45.6	0.71	(0.28, 1.79)	0.465
Male	33	55.9	1.00			26	44.1	1.00			39	66.1	1.00		
**Hispanic**															
Yes	8	50.0	1.63	(0.48, 5.53)	0.435	3	18.7	0.56	(0.12, 2.66)	0.468	10	62.5	0.87	(0.26, 2.92)	0.821
No	38	38.0	1.00			27	27.0	1.00			55	55.0	1.00		
**Race**															
Black	17	28.8	0.48	(0.19, 1.18)	0.110	11	18.6	0.63	(0.20, 1.92)	0.415	27	45.8	0.53	(0.23, 1.26)	0.152
Non-Black	29	50.9	1.00			19	33.3	1.00			38	66.7	1.00		
**Age**															
Mean (SD)	46	31.6 (7.86)	0.94	(0.89, 0.99)	**0.032**	30	32.3 (8.00)	0.98	(0.92, 1.05)	0.581	65	34.3 (10.08)	1.01	(0.96, 1.05)	0.782
**Household income**															
≤$50,000	25	29.8	0.46	(0.16, 1.37)	0.163	12	14.3	0.32	(0.10, 1.06)	0.063	41	48.8	0.61	(0.20, 1.83)	0.375
>$50,000	21	65.6	1.00			18	56.2	1.00			24	75.0	1.00		
**Nicotine dependence (PROMIS total score)**															
Mean (SD)	46	13.4 (4.45)	1.19	(1.06, 1.34)	**0.003**	30	14.8 (4.00)	1.24	(1.08, 1.43)	**0.002**	65	13.0 (4.37)	1.23	(1.09, 1.38)	**0.001**
Used another tobacco product* **in past 30 days**															
Yes	38	44.7	1.87	(0.60, 5.80)	0.277	26	30.6	1.56	(0.37, 6.61)	0.543	48	56.5	0.50	(0.17, 1.47)	0.210
No	8	25.8	1.00			4	12.9	1.00			17	54.8	1.00		

* E-cigarette, cigar, hookah, smokeless tobacco, nicotine pouch.

aOR = Adjusted Odd’s Ratio; SD= Standard Deviation **Bolded** = p-value < 0.05.

## Data Availability

Data will be made available on request.

## Appendix A. Supplementary data

Supplementary data to this article can be found online at 10.1016/j.addbeh.2025.108470.
