# Supplementary material for: Menthol cigarette alternative products: Awareness, use, and substitution intentions among adults who smoke menthol cigarettes
Source: Addict Behav. Author manuscript; Available in PMC 2025 Dec 15. (PMC12703848; doi:10.1016/j.addbeh.2025.108470)

**Supplementary Material. Descriptions and images of alternative menthol cigarette products.**

**Other Menthol Products**

There are several products on the market that people may use to add menthol or menthol flavor to regular non-menthol cigarettes. We include brief descriptions and images of several of these products. Please read each product description carefully and answer the questions below for each.

**Menthol filter capsules,** also known as crush balls or flavor popping beads. These are small pre-filled capsules that you buy separately from cigarettes. You use a small tool to push the menthol-flavored ball into the filter of the cigarette. Once you are ready for the menthol flavor, you crush the ball.


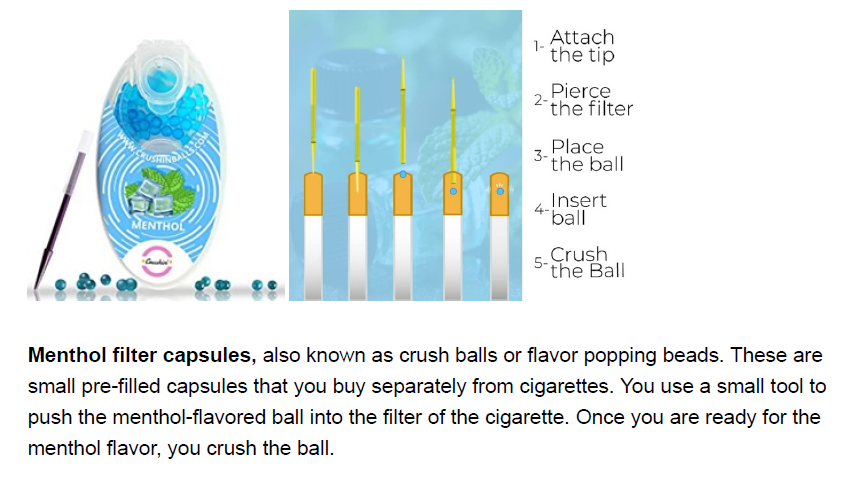


**Menthol spray.** This product is sprayed directly onto the filters of regular cigarettes and must absorb for a period of time (usually 2 hours or more) to give the cigarette a menthol flavor.

**
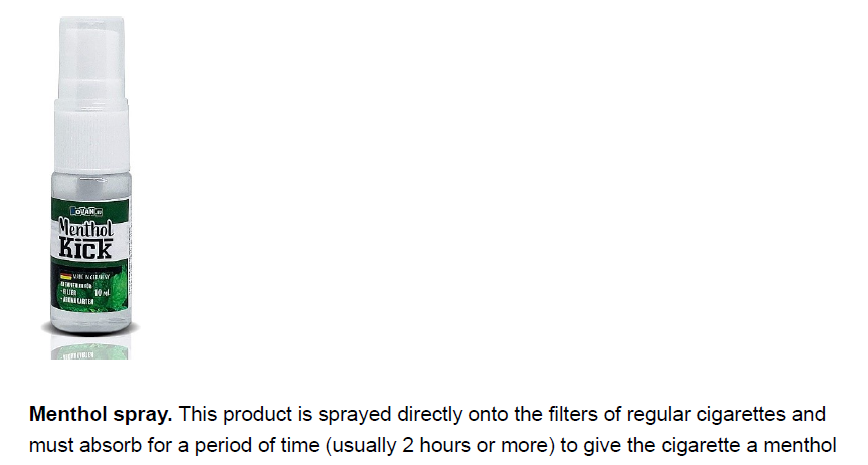
**

**Menthol flavor cards.** These are small paper cards, about the size of a pack of cigarettes, that are infused or covered with menthol flavor. The card slides into a pack of cigarettes and can be left in the pack. The cigarettes in the pack absorb the menthol flavor over time.

**
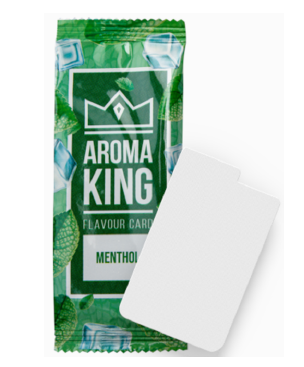
**

**Menthol filter tips.** These are menthol-flavored filters that are used for rolling your own cigarettes. They do not work with pre-made cigarettes that you can buy at the store. The filters may or may not contain menthol flavor capsules (like crush balls).


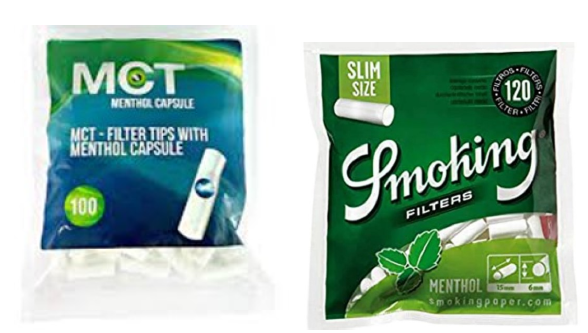


**Non-menthol cooling cigarettes.** Menthol cigarettes are already banned in some places, like California and Massachusetts. Some tobacco companies have added other cooling chemicals to their cigarettes instead of menthol. These cigarettes are labeled as non-menthol (because they do not contain menthol flavor), and have other cooling flavors instead. They are sometimes called "crisp" or "fresh" non-menthol cigarettes.


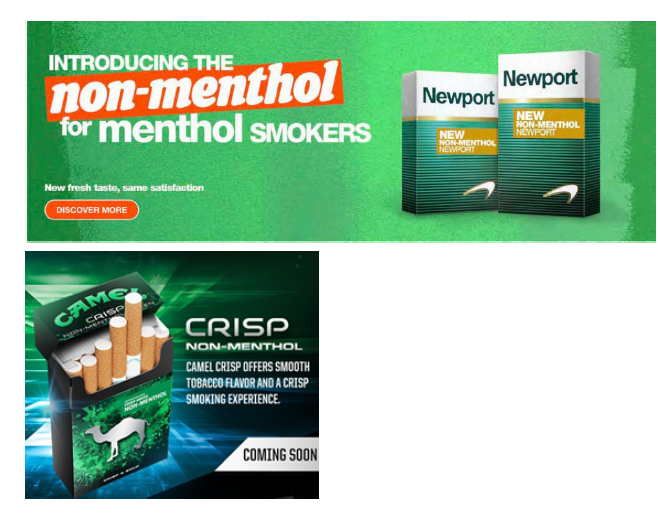

Supplement: MMC1 [file NIHMS2107587-supplement-MMC1.docx]
